# A124 CHARACTERISTICS OF GASTRIC CANCER IN BRITISH COLUMBIA

**DOI:** 10.1093/jcag/gwae059.124

**Published:** 2025-02-10

**Authors:** S Sasson, D Farnell, D Motomura

**Affiliations:** University of British Columbia, Department of Medicine, Vancouver, BC, Canada; Department of Pathology and Laboratory Medicine, University of British Columbia, Vancouver, BC, Canada; Department of Gastroenterology, University of British Columbia, Vancouver, BC, Canada

## Abstract

**Background:**

Gastric cancer remains a significant global health concern. In Asia, the prevalence of H. Pylori (HP) infection has led to well described risk factors in a more homogenous populace. In contrast, British Columbia presents a more diverse population, yet there is limited data on the specific characteristics of gastric cancer in this region.

**Aims:**

1. Compare the distribution and characteristics of histological types of gastric cancer using both the Lauren and Japanese classification.

2. Compare early-stage gastric cancers (T1) and advanced stages, focusing on the effectiveness of early detection and treatment approaches.

**Methods:**

We conducted a retrospective analysis of patients diagnosed with gastric cancer between 2012 and 2024 from 5 major centers in the Greater Vancouver area. This includes patients from the interior of British Columbia. Patients with gastroesophageal junction (GEJ) or gastric cardia lesions were excluded.

For each patient, demographic data (including ethnicity), lesion characteristics, and risk factors were collected. HP positivity was confirmed by histological inspection or a prior positive non-invasive test. Data were analyzed using standard stastical methods.

**Results:**

The initial search identified 297 patients, with 167 meeting the inclusion criteria (mean age 68 years, male 62%, diffuse histology 46%, surgical resection 93%, H. pylori positive 40%). Patients with intestinal-type gastric cancer were more likely to be male (77% vs. 49%), older (mean age 72 years vs. 63 years), and had higher rates of background atrophic gastritis (68% vs. 34%). Intestinal-type patients were more likely to meet criteria for endoscopic resection (17% vs. 2%). There were no significant differences in ethnicity or HP status between the histological subtypes. Tumor size approached significance, with intestinal-type cancers tending to be smaller (3.9 cm vs. 5.0 cm, p = 0.067). Early gastric cancer represented 22% of the cohort. Among these, 41% met criteria for endoscopic resection, though only 3 patients actually underwent the procedure (21% of those eligible).

**Conclusions:**

Gastric cancers in British Columbia are being diagnosed at a more advanced stage compared to some Asian populations, specifically in Japan. A significant proportion of patients eligible for endoscopic resection are instead undergoing surgery. Intestinal-type gastric cancers are more likely to be endoscopically resectable, likely due to more liberal resection. Patients with intestinal-type gastric cancer are also more likely to have background changes in the stomach, indicating a need for closer follow-up when this is detected. No significant ethnic differences were observed between histological subtypes.

Our findings underscore the need for improved early detection strategies in British Columbia, as well as the potential to reduce unnecessary surgeries through increased use of endoscopic resection.

**Table.** Comparison of Clinicopathological Features of Gastric Cancer Subtypes Based on Histological Type (Intestinal, Diffuse, Mixed) and Differentiation Status.

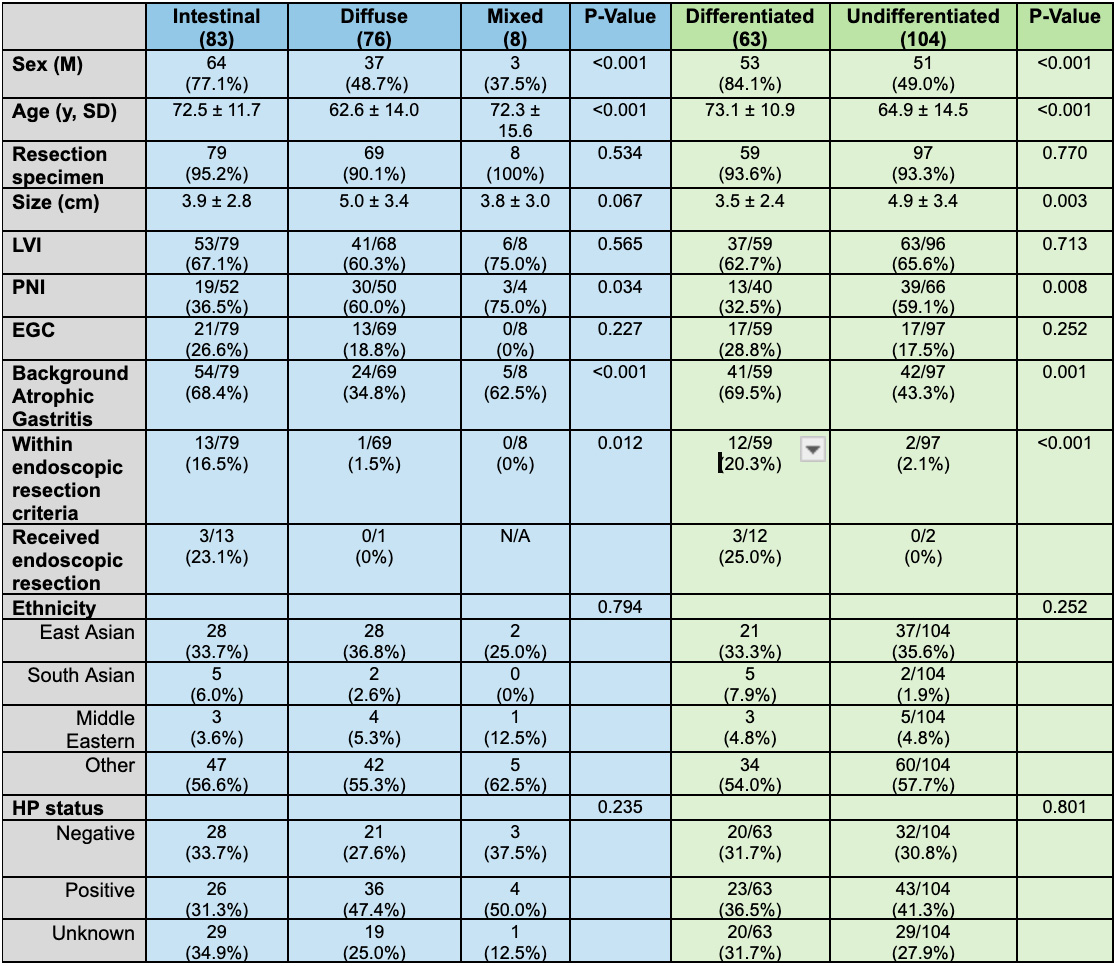

**Funding Agencies:**

None

